# Reply to the letter to the editor regarding “A prospective randomized study of multimodal analgesia combined with single injection transversus abdominis plane block versus epidural analgesia against postoperative pain after laparoscopic colon cancer surgery”

**DOI:** 10.1007/s00384-024-04616-9

**Published:** 2024-04-16

**Authors:** Tatsuya Manabe, Hiroshi Kitagawa, Yasutaka Yamada, Yoshiro Sakaguchi, Hirokazu Noshiro

**Affiliations:** 1 Department of Surgery, Faculty of Medicine, Saga University, 5-1-1 Nabeshima, Saga, Saga 849-8501 Japan; 2 Department of Anesthesiology, Faculty of Medicine, Saga University, 5-1-1 Nabeshima, Saga, Saga 849-8501 Japan

Dear Editor:

We appreciate Dr. Cheng-Wen Li and his colleagues for their interest in our article [1] and also appreciate them for information about recent studies. We would like to address each of four points that they raised.

First, they pointed out patient’s status at pain assessment in our study. In this study, a visual analog scale (VAS) was recorded by the nursing staff every morning when patients lay at rest in the bed before moving. As they pointed out, pain during activity must be more severe and the important factor for assessment of postoperative analgesia. As shown in the “Method” section, we set the use of additional analgesics until postoperative day 2 as the primary endpoint, which could be suitable for pain-associated evaluation with a small number of patients. We thought that a VAS would not differ between two groups in this study, and pain at rest was easy to record by nursing staffs. Therefore, we evaluated only a VAS of pain at rest.

Second, regarding concerns about the design of this study, we agree that the analgesic protocol without regular administration of paracetamol in epidural analgesia (EA) group might not be suitable for recommendation of Enhanced Recovery After Surgery society and that multimodal analgesia has become gold standard regardless of the use of thoracic epidural analgesia [2]. However, as a matter of fact, the analgesic procedure in the EA group has been the standard analgesic in our region. Therefore, that protocol was adapted as the control group.

Third issue was that flurbiprofen axetil could be insufficient for postoperative pain of a VAS of > 4 as an additional analgesic. Flurbiprofen axetil is a cyclo-oxygenase inhibitor that blocks the formation of prostaglandins and provides effective analgesia after surgery [3, 4]. In this study, additional analgesics (flurbiprofen axetil 50 mg) were administered not only when a VAS was > 4, but when the patients requested. Therefore, in our protocol, the patients could be administered with a VAS < 4. On the other hand, the frequency of additional analgesics was almost one, and repeated additional analgesics a day was rare in this study. Therefore, we think that flurbiprofen axetil has an analgesic effect on patients even with a VAS of > 4.

The final issue was validity of use of fentanyl. As they pointed out, fentanyl could cause opioid-related adverse effects on postoperative recovery. Although transversus abdominis plane block (TAPB) is an effective regional analgesia, it has not been demonstrated that TAPB is superior to EA on analgesic effect until now [5]. Therefore, fentanyl in addition to TAPB was used as basement analgesia in the multimodal analgesia group. As a result, a VAS was comparable between two groups, and the frequency of additional analgesics decreased in the multimodal analgesia group on POD2. Moreover, in the EA group, fentanyl was also administered via epidural route. Therefore, we believe that the postoperative course, such as bowel movement and hospital stay, was comparable between two groups.

## Data Availability

No datasets were generated or analyzed during the current study.
